# Polymorphism and molecular characteristics of the *CSN1S2* gene in river and swamp buffalo

**DOI:** 10.5194/aab-63-345-2020

**Published:** 2020-09-18

**Authors:** Xinyang Fan, Shanshan Gao, Lin Fu, Lihua Qiu, Yongwang Miao

**Affiliations:** 1 Faculty of Animal Science and Technology, Yunnan Agricultural University, Kunming 650201, Yunnan, China

## Abstract

The $\alpha_{S2}$-casein ($\alpha_{S2}$-CN) is a member of the casein family associated with milk traits in ruminants, but so far the buffalo *CSN1S2* gene has not been well understood. In this work, the polymorphisms of *CSN1S2* in river and swamp buffalo were detected using direct sequencing of polymerase chain reaction (PCR) products. As a result, 13 single nucleotide polymorphisms (SNPs) were identified in the coding sequence (CDS) of *CSN1S2* in two types of buffalo, of which eight SNPs were non-synonymous. The amino acid changes caused by c.580T>C and c.642C>G may affect the function of buffalo $\alpha_{S2}$-CN. A total of 11 *CSN1S2* CDS haplotypes were defined, and accordingly 11 variants of buffalo $\alpha_{S2}$-CN were inferred and named. The *CSN1S2* CDSs of both types of buffalo were 669 nucleotides, which encoded a precursor of 222 amino acids (AAs), and the first 15 AAs constitute a signal peptide. The composition and physicochemical characteristics of two types of buffalo $\alpha_{S2}$-CNs were similar but slightly different from those of cattle $\alpha_{S2}$-CN. The $\alpha_{S2}$-CN mature peptides of buffalo and the species of *Bos* genus contained a casein domain, and their secondary structures were highly consistent, indicating that they are functionally similar. The results here provide initial insights into the variation, characteristics and biological function of buffalo *CSN1S2*.

## 1 Introduction

Casein (CN) is the main protein in milk, accounting for about 80 % of milk protein, including $\alpha_{S1}$-, $\alpha_{S2}$-, β- and κ-CN. It can provide the suckling infant with amino acids, calcium and phosphate and is related to lactation traits of dairy animals and milk processing characteristics (Boettcher et al., 2004; Wedholm et al., 2006; Nilsen et al., 2009). The *CSN1S2* gene encodes $\alpha_{S2}$-CN, which accounts for 10 % of the total CN in the milk of dairy cows (Farrell et al., 2004). The $\alpha_{S2}$-CN, together with $\alpha_{s1}$-CN and β-CN, is called a calcium-sensitive protein which can form stable micelles with calcium and phosphorus to support bone growth in the young (Lefèvre et al., 2009; Corral et al., 2013). In addition, the *CSN1S2* gene is related to the nutritive value of milk (Wedholm et al., 2006).

The cattle* CSN1S2 *gene has been mapped in chromosome 6 and contains 18 exons, of which exon 1 is located in the 5${}^{\prime }$ untranslated region (UTR), exon 2 encodes the signal peptide from the 13th nucleotide, exons 3–16 are located in the coding sequence (CDS) region and encode a mature peptide, and exons 17–18 are in the 3${}^{\prime }$ UTR. The CDS length is 669 bp and encodes a protein consisting of 222 amino acids (AAs), of which the first 15 AAs form an N-terminal signal peptide (Farrell et al., 2004). The $\alpha_{S2}$-CN variants in the *Bos* genus have been widely studied for many years. So far, five $\alpha_{S2}$-CN variants (A, B, C, D and E) have been identified in the *Bos* genus (Caroli et al., 2009; Gallinat et al., 2013). Among them, variant A is the most prevalent and is regarded as the reference protein. Only two $\alpha_{S2}$-CN alleles, A and B, have been reported in buffalo (Cosenza et al., 2009).

As a kind of dairy, meat and draft animal, the water buffalo (*Bubalus bubalis*) has important economic value in countries of tropical and subtropical areas (Michelizzi et al., 2010). According to the morphological and behavioral criteria, domestic buffalo can be divided into swamp buffalo and river buffalo. The former is mainly used for draft, while the latter is mainly used for milk production. In the past few years, buffalo have provided about 13 % of the total milk production in the world (Basilicata et al., 2017). In addition, buffalo milk has physicochemical characteristics different from cow milk. Compared with the milk of dairy cattle, buffalo milk has a higher content of total solids, fat and protein (Ahmad et al., 2013). Similarly, buffalo milk has been identified as containing the $\alpha_{S2}$-CN component encoded by the *CSN1S2* genes which are located on chromosome 7 (Iannuzzi et al., 2003). Up to now, little research has been done on the *CSN1S2* gene in buffalo at the molecular level. In view of the lack of information about this gene, we detected the single nucleotide polymorphisms (SNPs) in the CDS of the *CSN1S2* gene for two types of buffalo using direct sequencing of polymerase chain reaction (PCR) products. The $\alpha_{S2}$-CN variants in two types of buffalo were characterized, and the differences of $\alpha_{S2}$-CN variants between buffalo and the species of *Bos* genus were further investigated. The results can provide a basis for revealing the molecular characteristics, function and variation of the buffalo *CSN1S2* gene.

## 2 Materials and methods

### 2.1 Animal source and sample collection

Ear tissue samples were obtained from 120 river buffalo (74 Binglangjiang, 32 Murrah and 14 Nili-Ravi buffalo) and 284 swamp buffalo (58 Xilin, 42 Yanjin, 72 Dechang, 52 Fuzhong and 60 Guizhou buffalo). The buffalo used for sample collection were all healthy adult buffalo without direct kinship. To further align with buffalo, the published CDS sequences of the *CSN1S2* gene in the *Bos* genus including *Bos taurus*, *Bos grunniens*, *Bos mutus* and *Bison bison* in the National Center for Biotechnology Information (NCBI) database (https://www.ncbi.nlm.nih.gov/, last access: 10 May 2020) were downloaded and used for data analysis in this study.

All procedures for sample collection were performed in accordance with the Guide for Animal Care and Use of Laboratory Animals approved by the Yunnan Provincial Experimental Animal Management Committee under contract 2007-0069.

### 2.2 DNA extraction, PCR and sequencing

Genomic DNA was isolated from the ear tissue following a protocol described by Sambrock and Russell (2001). Their quality was detected by using 1.5 % agarose gel and further quantified using a NanaDrop LITE spectrophotometer (Thermo Fisher Scientific, USA). Subsequently, six pairs of primers were designed to amplify the CDS of the buffalo *CSN1S2* gene according to the genome sequence of buffalo *CSN1S2* (accession no. NC_037551) by Primer Premier 5.0 (Table 1) (Lalitha 2000). The 25 µL reaction system consisted of 0.6 µM of each primer, 100 ng of DNA template and 12.5 µL of 2× GoldStar MasterMix (dye) (CWBio, China). The PCR protocol was performed according to the manufacturers' instructions of 2× GoldStar MasterMix (dye). Then the amplified product was electrophoresed on agarose gel, and the target band was purified by cutting gel recovery and further sequenced bidirectionally using the Sanger method.

**Table 1:** Primer information for PCR and polymorphism identification.

Amplified	Primer sequence (5${}^{\prime }$ to 3${}^{\prime }$)	Products	Annealing	Extension
region		length (bp)	temperature (${}^{\circ }$C)	time (s)
Exon 2	F: TATGCCCAAATGAGCCTCCA	427	53	30
	R: TCCCTCTCTATTCCCTGCTGTC			
Exon 3–5	F: TGCCATCAAAACAAACAGGA	1279	50	105
	R: TGTGGCTCAAAAATGGCTC			
Exon 6–8	F: TTGAGAGCCATTTTTGAGCC	1628	51	125
	R: GCTCACCCTATTTGCGATGT			
Exon 9–12	F: AATGAATTGCCCTTTCTACTC	1369	52.5	105
	R: TTCCCCAGATTTTTCTTAGG			
Exon 13	F: GCATTTAGCCAGCATTATG	220	50	25
	R: ATCTTACCATGTCAACGGTCT			
Exon 14–16	F: TTACTGGTGGGCTATTCAAGT	1584	52.5	120
	R:CAATTTCCAGCCTAGAACATTC			

### 2.3 Sequence data analysis

The obtained sequences of buffalo *CSN1S2* were compared, proofread and edited through the Lasergene software package (DNASTAR Inc., USA). Mutation sites were exported with MEGA 6 (Tamura et al., 2013), and estimation of allele and genotype frequencies and Hardy–Weinberg equilibrium test were carried out adopting PopGen32 (Yeh and Boyle, 1997). The function influence of non-synonymous substitutions was presumed using PROVEAN (http://provean.jcvi.org/index.php, last access: 10 May 2020). The haplotypes were analyzed by PHASE (Stephens et al., 2001). The genetic relationship among the haplotypes was constructed by Network 5 (http://www.fluxus-engineering.com, last access: 8 May 2020; Bandelt et al., 1999). The physicochemical characteristics, signal peptide and subcellular localization of buffalo $\alpha_{S2}$-CN were predicted using the ProtParam tool (http://web.expasy.org/protparam/, last access: 10 May 2020), SignalP 5.0 server (http://www.cbs.dtu.dk/services/SignalP/, last access: 10 May 2020) and ProtComp 9.0 (http://linux1.softberry.com/berry.phtml, last access: 10 May 2020), respectively. The phosphorylation site was presumed through the NetPhos 3.1 Server (http://www.cbs.dtu.dk/services/NetPhos/, last access: 8 May 2020). The conserved domains of buffalo $\alpha_{S2}$-CN were ascertained through the Conserved Domain Architecture Retrieval Tool in BLAST (http://www.ncbi.nlm.nih.gov/BLAST, last access: 10 May 2020). The inferred secondary structure of amino acid sequence was determined by SOPMA (http://npsa-pbil.ibcp.fr/, last access: 10 May 2020).

## 3 Results

### 3.1 Polymorphism analysis of the buffalo *CSN1S2* gene

**Figure 1:**
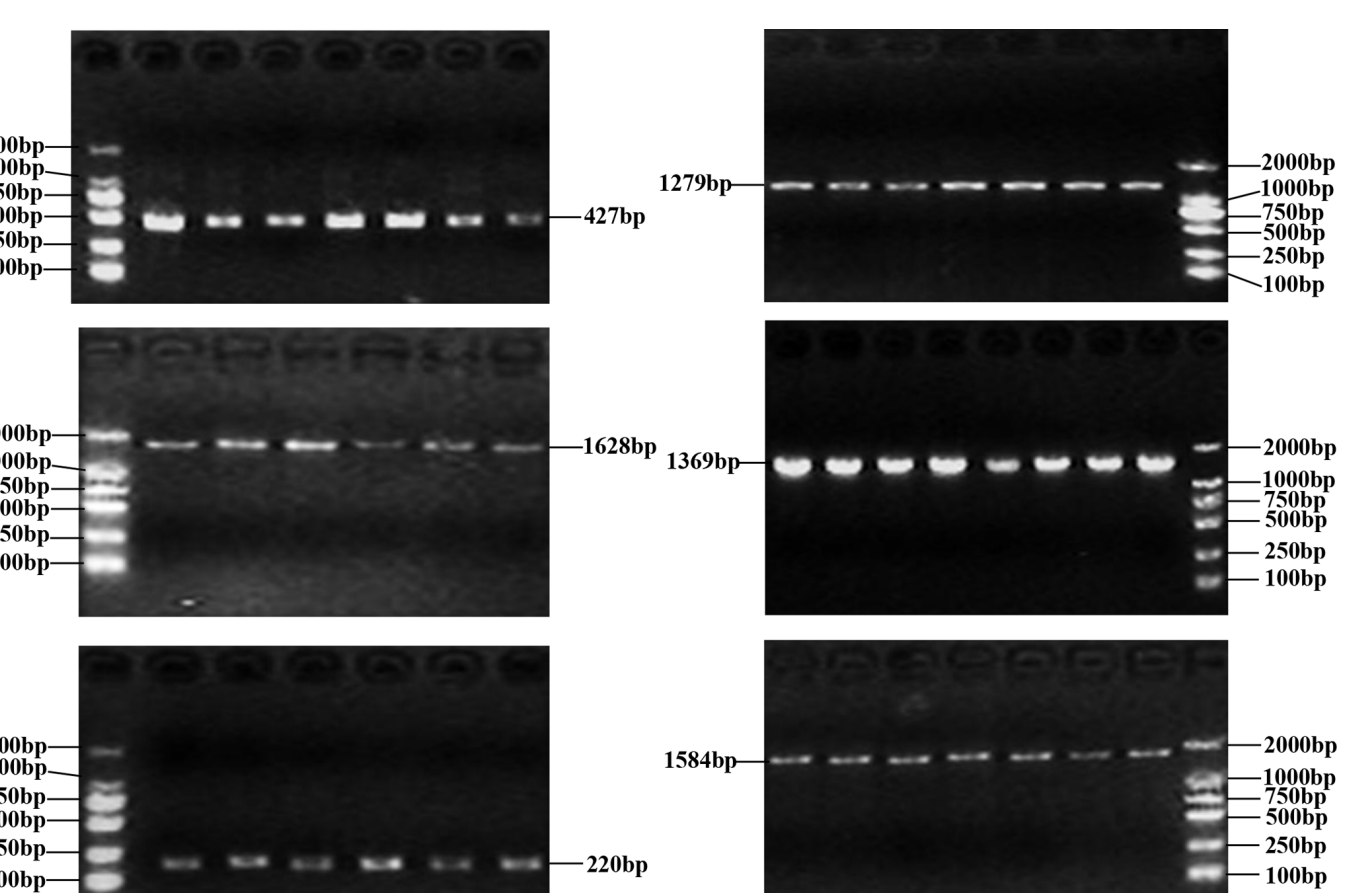
Six PCR-amplified fragments of buffalo *CSN1S2* detected by agarose gel electrophoresis.

The PCR products as expected were obtained (Fig. 1). The obtained sequences were assembled and confirmed by comparing them with the homologous sequences of the *Bos* genus published in NCBI database. In the samples of this work, three SNPs were found in the buffalo *CSN1S2* gene, in which c.234C>A was located in exon 9, c.391G>A in exon 11 and c.568G>A in exon 16 (Table 2) with the exception that c.568G>A was only found in river buffalo and the other SNPs were shared by two types of buffalo. It is noteworthy that allele frequency in river buffalo at SNP234 was markedly different from that in swamp buffalo. SNP234 was nearly homozygous in river buffalo, but the heterozygote frequency was still high in swamp buffalo. The test for Hardy–Weinberg equilibrium showed that only SNP234 in swamp buffalo was in disequilibrium (P<0.05) (Table 2), indicating that the SNP may be affected by selection and genetic drift.

**Table 2:** Polymorphic loci and their allelic and genotypic frequencies in two types of buffalo.

Population	SNP	Genotype		Allele		P value${}^{*}$
		frequency		frequency		
River buffalo	c.234A>C	AA	0.983	A	0.9915	1.00000
		AC	0.017	C	0.0085	
		CC	0.000			
	c.391G>A	GG	0.948	G	0.9741	0.86943
		GA	0.052	A	0.0259	
		AA	0.000			
	c.568G>A	GG	0.339	G	0.5893	0.85779
		GA	0.500	A	0.4107	
		AA	0.161			
Swamp buffalo	c.234A>C	AA	0.248	A	0.4204	0.00164
		CA	0.345	C	0.5796	
		CC	0.407			
	c.391G>A	GG	0.558	G	0.7458	0.86842
		GA	0.375	A	0.2542	
		AA	0.067			

${}^{*}$ P value of Hardy–Weinberg equilibrium test.

Having pooled the data of this work with published buffalo sequences (accession numbers FM865618, DW007991, DW007964, DW007983, FM865619 and DQ173244) in the NCBI database, 10 additional SNPs were found in river buffalo, i.e., c.15T>C, c.381T>A, c.382A>G, c.459C>T, c.484T>A, c.580T>C, c.587A>G, c.618G>A, c.627T>C and c.642C>G, and the number of polymorphic sites increased to 13. Among them, c.234C>A, c.382A>G, c.391G>A, c.484T>A, c.568G>A, c.580T>C, c.587A>G and c.642C>G were non-synonymous, leading to changes in p.Glu63Asp, p.Lys113Glu, p.Ala116Thr, p.Phe147Ile, p.Ala175Thr, p.Tyr179His, p.Lys181Arg and p.Asn199Lys in the mature peptide (Table S1 in the Supplement). The prediction showed that the substitutions of p.Tyr179His and p.Asn199Lys probably affected the function of $\alpha_{S2}$-CN.

### 3.2 Haplotype inference and their genetic relationship

**Figure 2:**
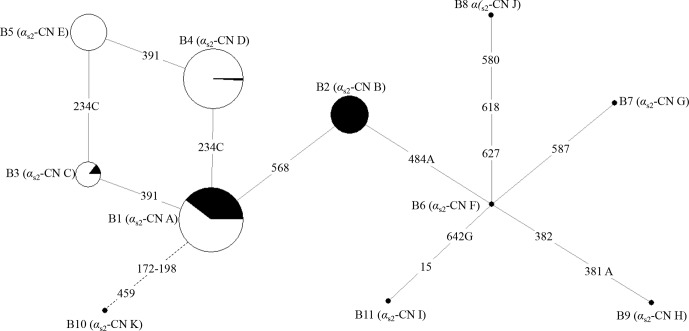
Network of 11 haplotypes of the buffalo *CSN1S2* gene. B1–B11 are the haplotypes defined here in buffalo. Mutations along the branch are labeled by the nucleotide positions in the CDS, and transversions are specified by the further addition of suffixes A, G and C. The dotted line represents the haplotype generated from skipping exon 7 after transcription. Each haplotype is represented by a circle with the area of the circle proportional to its frequency. Samples from river and swamp buffalo are indicated by black and white color, respectively.

**Figure 3:**
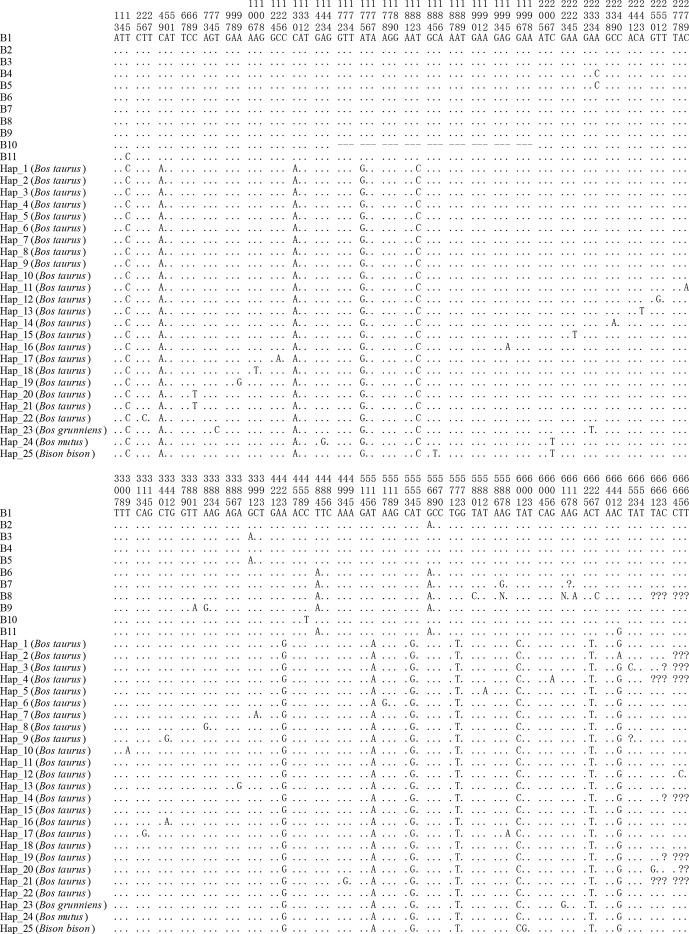
Sequence difference sites of the haplotypes between buffalo and bovine. The corresponding accession numbers of 25 haplotype sequences (Hap1 to Hap25) of the *Bos* genus are XM_024993017, DR712148, DR711516, DT854174, DR711908, DT849277, DR712290, DR711611, DR711989, EH123804, DR711761, DR71135, M16644, DR711495, DR711941, DR711568, BC114773, DR711392, DR711419, BG690139, BG691827, DR711347, MH378279, XM_014480230 and XM_010852145, respectively. Numbers represent the position in the CDS. Dots (.) represent the identity with the haplotype B1. Nucleotide substitutions are denoted by different letters. Horizontal lines (–) represent the deletion in the sequences. Missing information is denoted by the question marks (?).

According to the SNPs in the CDS of the buffalo *CSN1S2* gene, a total of 11 haplotypes (B1–B11) were inferred in two types of buffalo (Figs. 2 and 3). Among them, five (B1–B5) (accession numbers MT316464–MT316468) were obtained from the data of this study (Table 3), and the other six were from published data (accession numbers FM865618, DW007991, DW007964, DW007983, FM865619 and DQ173244). Among these haplotypes, B1, B3 and B4 were shared by two types of buffalo, B5 was found only in swamp buffalo, and the rest were only found in river buffalo. B6 and B10 (deletion of 27 nucleotides) were equivalent to the previously reported alleles A and B (Cosenza et al., 2009).

**Table 3:** Frequencies of *CSN1S2* haplotypes in two types of buffalo.

Haplotype	Base composition	Actual	Expected	AFR	AFS
ID	of haplotype	frequency	frequency		
B1	AGG	0.4356	0.4070	0.5833	0.3732
B2	AGA	0.1139	0.1183	0.3833	0.0000
B3	AAG	0.0520	0.0831	0.0250	0.0633
B4	CGG	0.2921	0.2932	0.0084	0.4121
B5	CAG	0.1064	0.0982	0.0000	0.1514

Note that the frequency is estimated by the program PHASE. AFR signifies actual frequency in river buffalo, and AFS signifies actual frequency in swamp buffalo.

The possible genetic relationships among these haplotypes of buffalo *CSN1S2* were investigated through employing median-joining network (Fig. 2). Haplotype B1 was the dominant haplotype which was widely distributed in two types of buffalo. Other haplotypes may originate from B1, which is to say that B2, B3 and B10 may evolve from B1 through one transition and B4 through one transversion. B5 may evolve from B4 through one transition or B3 through a transversion from A to C. B6 may evolve from B2 through a transversion from T to A. B7, B8, B9 and B11 may evolve from B6 through transitions and/or transversions. Whether this is the case or not, further experiments are needed to verify it.

### 3.3 Nucleotide differences of the haplotypes between buffalo and the species of *Bos* genus

All the haplotype sequences of the *Bos* genus published were downloaded and compared with this work in order to explore the differences in the CDS of the *CSN1S2* gene between buffalo and the species of *Bos* genus. A total of 25 haplotypes were defined in the *Bos *genus (Fig. 3). The comparison results showed that there were 10 differences in the all haplotypes between buffalo and the species of *Bos* genus, which were located at positions c.49, c.130, c.175, c.183, c.423, c.516, c.554, c.572, c.601 and c.626.

### 3.4 Nomenclature of buffalo $\alpha_{S2}$-CN variants

**Figure 4:**
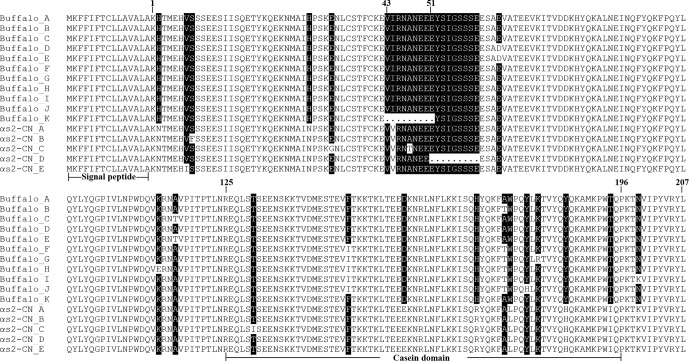
Sequence differences of $\alpha_{S2}$-CN variants between buffalo and the species of *Bos* genus. $\alpha_{S2}$-CN_A–E are the identified variant sequences in the *Bos* genus. Dots (.) represent the deletion in the sequences. Amino acid differences are denoted by black shading.

A total of 11 $\alpha_{S2}$-CN variants were identified in view of the haplotype sequences of buffalo *CSN1S2*. According to the existing nomenclature of the *Bos* genus, we named buffalo $\alpha_{S2}$-CN variants as A, B, C, D, E, F, G, H, I, J and K (Table 4). Sequence alignment showed that all the variants of the *Bos* genus identified previously have not been found in two types of buffalo, and there are eight amino acid differences between buffalo $\alpha_{S2}$-CN variants and bovine $\alpha_{S2}$-CN variants (Fig. 4), which include p.His2Asn, p.His29Asn, p.Ile44Val, p.Asp157Glu, p.His170Arg, p.Trp176Leu, p.Tyr186His and p.Thr194Ile. The sequence consistency of the $\alpha_{S2}$-CN variants among buffalo and the species of *Bos* genus was more than 93.0 % (Fig. S1 in the Supplement). In the samples of this work, the frequencies of the variants A, B, C and D in river buffalo were 58.3 %, 38.3 %, 2.5 % and 0.8 %, and the frequencies of the variants A, C, D and E in swamp buffalo were 37.3 %, 6.3 %, 41.2 % and 15.1 %, respectively.

**Table 4:** Amino acid positions and differences in genetic variants of buffalo $\alpha_{S2}$-CN.

Position${}^{*}$	$\alpha_{S2}$-CN variant (haplotype)										
	A (B1)	B (B2)	C (B3)	D (B4)	E (B5)	F (B6)	G (B7)	H (B9)	I (B11)	J (B8)	K (B10)
43–51											deleted
63	E GAA			D GAC	D GAC						
112	V GTT							GTA			
113	K AAG							E GAG			
116	A GCT		T ACT		T ACT						
138	T ACC										ACT
147	F TTC					I ATC	I ATC	I ATC	I ATC	I ATC	
175	A GCC	T ACC				T ACC	T ACC	T ACC	T ACC	T ACC	
179	Y TAT									H CAT	
181	K AAG						R AGG				
191	K AAG									AAA	
194	T ACT									ACC	
199	N AAC								K AAG		

${}^{*}$ Numbers represent the position of the mature peptide.

### 3.5 Molecular characteristics analysis

The length of *CSN1S2* CDS for two types of buffalo was 669 nucleotides, encoding a precursor peptide consisting of 222 AAs. Buffalo $\alpha_{S2}$-CN had a signal peptide of 15 AAs and a mature peptide of 207 AAs. The AA composition of buffalo $\alpha_{S2}$-CN variant A is shown in Table S2. In order to clarify the characteristics of the buffalo *CSN1S2* gene and their differences between buffalo and the species of *Bos* genus, we analyzed the molecular characteristics of major $\alpha_{S2}$-CN variants of buffalo (buffalo variant A from this study, accession no. MT316464) and cattle (variant A; accession no. M94327; Caroli et al., 2009) by bioinformatics methods. The AA composition and basic molecular characteristics of buffalo $\alpha_{S2}$-CN variant A were slightly different from those of cattle $\alpha_{S2}$-CN variant A (Table 5). Their mature peptides all contained a casein domain (from the AA125 to AA196) (Fig. 4), which belonged to the casein family. The secondary structure of buffalo variant A and cattle variant A were also similar (Fig. S2). There were only a few differences between them. The prediction of subcellular localization showed that both buffalo and cattle $\alpha_{S2}$-CNs were secreted extracellularly with high reliability (100 %).

**Table 5:** Basic physicochemical properties of mature $\alpha_{S2}$-CN peptides from buffalo and cattle.

	Buffalo A	Cattle A
Formula	$C_{1091}H_{1695}N_{287}O_{339}S_{6}$	$C_{1083}H_{1707}N_{287}O_{338}S_{6}$
Number of amino acids	207	207
Molecular weight	24.45 KD	24.35 KD
Isoelectric point (pI)	7.14	8.34
Strongly acidic amino acid (D, E)	28	28
Strongly basic amino acid (K, R)	28	30
Polar amino acid (N, C, Q, S, T, Y)	77	76
Hydrophobic amino acid (A, I, L, F, W, V)	53	54
Instability index (II)	45.64	46.27
Grand average of hydropathicity (GRAVY)	-0.944	-0.918
Aliphatic index	65.41	68.70
Number of amino acids in signal peptide	15	15
Number of phosphorylation sites	30	29

## 4 Discussion

In recent years, the polymorphisms of milk proteins have aroused great research interest because the genotypes of milk proteins may be related to milk composition and milk yield of dairy cattle (Nilsen et al., 2009). In this work, the SNPs of the *CSN1S2* gene in two types of buffalo were investigated. As a result, 13 SNPs were determined in buffalo. The allele frequency of river buffalo at SNP234 was significantly different from that in swamp buffalo, and SNP568 found in river buffalo has been homozygous in swamp buffalo, which indicated that the variation of the *CSN1S2* gene in two types of buffalo had different population genetic characteristics. It was found that two non-synonymous substitutions in buffalo, i.e., c.580T>C and c.642C>G, led to amino acid changes in p.Tyr179His and p.Asn199Lys which seriously affected the function of the $\alpha_{S2}$-CN. The Tyr and Asn are polar neutral AAs, while the His and Lys are basic AAs. These substitutions belong to substitutions with different physicochemical properties, suggesting that they may cause changes in the structure or function of buffalo $\alpha_{S2}$-CN. Whether the SNPs identified in this study, especially the non-synonymous SNPs, have any influence on the function of $\alpha_{S2}$-CN and lactation traits of buffalo needs to be further verified by the association with lactation trait data.

In recent years, the $\alpha_{S2}$-CN variants of the *Bos* genus have been determined and named (Gallinat et al., 2013). Nevertheless, due to the limited previous studies on the polymorphism of the buffalo *CSN1S2* gene, the nomenclature of $\alpha_{S2}$-CN variants in buffalo has not been fully developed. In this work, we analyzed the polymorphisms of the *CSN1S2* gene in two types of buffalo to achieve a full understanding of buffalo $\alpha_{S2}$-CN variants. It is necessary to separately define the variants of buffalo $\alpha_{S2}$-CN due to the large sequence differences of the *CSN1S2* gene between buffalo and the species of *Bos* genus. We named 11 variants in buffalo $\alpha_{S2}$-CN based on the nomenclature convention based on buffalo *CSN1S2* haplotypes. In previous studies, it has been reported that there are two kinds of *CSN1S2* transcripts in river buffalo, one covering seven exons with a CDS length of 669 bp and the other having an exon 7 skipping (variant K in this work) with a CDS length of only 642 bp (Cosenza et al., 2009). In this work, we screened SNPs at the DNA level; thus, only the former *CSN1S2* transcript was identified in both types of buffalo. It is worth noting that the $\alpha_{S2}$-CN variants with the highest frequency in the two types of buffalo were different. The distribution frequency of variant A is the highest in river buffalo, while the distribution frequency of variant D is the highest in swamp buffalo.

According to the network of buffalo *CSN1S2* haplotypes, $\alpha_{S2}$-CN variants B–D differ from variant A by one AA variation, and they may directly evolve from variant A. Buffalo variant E may evolve from variant C or D through one AA exchange, and variant F may evolve from variant B through one AA exchange. Buffalo variants G, H, I and J evolved from variant F through one AA exchange for variant G, one AA exchange with one synonymous substitution for H, one AA exchange for I, and one AA exchange with two synonymous substitutions for J. Variant K may directly originate from A through exon 7 skipping with one synonymous substitution. Haplotypes B6–B11 came from the sequences published in the database. It is worth noting that the variants encoded by these sequences are very different from those determined by the samples in this study.

Previous studies have confirmed that the *CSN1N2* gene exerts an important function in milk traits. In cattle, studies have revealed that the *CSN1S2* gene is closely related to the milk protein (Aleandri et al., 1990; Ikonen et al., 2001). The variation in the *CSN1S2* gene is associated with the synthesis rate of $\alpha_{S2}$-CN in milk (Ibeagha-Awemu et al., 2008). In addition, the function of $\alpha_{S2}$-CN is related to the formation of CN micelles (Johansson et al., 2009). In this study, the $\alpha_{S2}$-CN mature peptides for both river and swamp buffalo were all composed of 207 AAs, and their basic physicochemical properties and amino acid composition were basically similar but slightly different from those of cattle. The influence of this difference on function needs to be further studied. It is well known that the lactation characteristics of river buffalo and swamp buffalo are distinct. Because the main variants of $\alpha_{S2}$-CN in the two types of buffalo are different, their physicochemical properties are also different. This difference may be one of the reasons for the difference in lactation characteristics between the two types of buffalo. This study showed that the buffalo $\alpha_{S2}$-CN mature peptide contains all 20 amino acids, of which essential amino acids account for 42.4 %, indicating that buffalo $\alpha_{S2}$-CN is one of the important sources of amino acids for the suckling calves. The $\alpha_{S2}$-CN mature peptides of buffalo and cattle have a casein domain with the same length and similar amino acid composition, and the percent identities of the sequences were also high, suggesting that the functions of $\alpha_{S2}$-CN in the two species are similar. The prediction showed that the secondary structure of buffalo $\alpha_{S2}$-CN was highly consistent with that of cattle $\alpha_{S2}$-CN. Considering the highly consistent molecular characteristics of $\alpha_{S2}$-CN between buffalo and cattle, it can be speculated that buffalo $\alpha_{S2}$-CN also fulfills crucial function in the formation of casein micelles.

Protein phosphorylation is a significant posttranscriptional modification which can regulate the structure and function of milk protein (Li et al., 2012). Phosphorylation is very important for the binding of metal ions to casein micelles. Casein micelle has an effect on milk coagulation and cheese making (Fan et al., 2019). Taken together, the phosphorylation of $\alpha_{S2}$-CN significantly affects the milk processing characteristics. Because the amino acid at position 194 in the mature peptide of buffalo $\alpha_{S2}$-CN was different from that of bovine $\alpha_{S2}$-CN (buffalo, 194Thr; cattle, 194Ile), the number of putative phosphorylation sites in buffalo was one more than in bovines. Consequently, there may be some differences in phosphorylation process of $\alpha_{S2}$-CN between buffalo and cattle, which may lead to differences in the structure and function. This may lead to differences in processing characteristics between buffalo milk and bovine milk.

## 5 Conclusions

In this work, 13 SNPs were determined in the buffalo *CSN1S2* gene. Among them, eight were non-synonymous substitutions. The *CSN1S2* gene of river and swamp buffalo had different population genetic characteristics. A total of 11 haplotypes were defined in the buffalo *CSN1S2* gene, and accordingly 11 $\alpha_{S2}$-CN variants were identified in buffalo. The amino acid composition and physicochemical characteristics of buffalo $\alpha_{S2}$-CN are slightly different from those of cattle. The functional domain and secondary structure of buffalo $\alpha_{S2}$-CN were similar to those of the *Bos* genus $\alpha_{S2}$-CN. Whether these SNPs have any influence on the milk traits of buffalo needs to be further studied.

## Supplement

10.5194/aab-63-345-2020-supplement The supplement related to this article is available online at: https://doi.org/10.5194/aab-63-345-2020-supplement.

## Data Availability

The original data used in this study are available from the corresponding author upon request.
