# Supplementary material for: Polymorphism and molecular characteristics of the CSN1S2 gene in river and swamp buffalo
Source: Arch Anim Breed. 2020 Sep 18;63(2):345–54. doi: 10.5194/aab-63-345-2020 (PMC8162411; doi:10.5194/aab-63-345-2020)
Supplement: The supplement related to this article is available online at: https://doi.org/10.5194/aab-63-345-2020-supplement. [file aab-63-345-supplement.pdf]

Supplement of Arch. Anim. Breed., 63, 345–354, 2020
https://doi.org/10.5194/aab-63-345-2020-supplement
© Author(s) 2020. This work is distributed under
the Creative Commons Attribution 4.0 License.

Open Access
Archives Animal Breeding

Supplement of

Polymorphism and molecular characteristics of the *CSN1S2* gene in river and swamp buffalo

Xinyang Fan et al.

Correspondence to: Yongwang Miao (yongwangmiao1@126.com)

The copyright of individual parts of the supplement might differ from the CC BY 4.0 License.

Table S1. The functional effect of non-synonymous substitutions on buffalo α_{S2}-CN

SNP	Substitution	PROVEAN score	Prediction (cutoff=-2.5)
c.234C>A	p.Glu63Asp	-0.301	Neutral
c.382A>G	p.Lys113Glu	-1.621	Neutral
c.391G>A	p.Ala116Thr	-1.693	Neutral
c.484T>A	p.Phe147Ile	-2.350	Neutral
c.568G>A	p.Ala175Thr	1.936	Neutral
c.580T>C	p.Tyr179His	-3.981	Deleterious
c.587A>G	p.Lys181Arg	-2.072	Neutral
c.642C>G	p.Asn199Lys	-2.602	Deleterious

Table S2. Amino acid composition of mature peptide of buffalo α_{S2}-CN variant A

Amino acids	Number	Frequency (%)	Amino acids	Number	Frequency
Ala (A)	8	3.9	Lys (K)	23	11.1
Arg (R)	5	2.4	Met (M)	4	1.9
Asn (N)	13	6.3	Phe (F)	6	2.9
Asp (D)	5	2.4	Pro (P)	10	4.8
Cys (C)	2	1.0	Ser (S)	17	8.2
Gln (Q)	16	7.7	Thr (T)	16	7.7
Glu (E)	23	11.1	Trp (W)	3	1.4
Gly (G)	2	1.0	Tyr (Y)	13	6.3
His (H)	5	2.4	Val (V)	13	6.3
Ile (I)	11	5.3	Pyl (O)	0	0.0
Leu (L)	12	5.8	Sec (U)	0	0.0

Percent Identity

Divergence

	1	2	3	4	5	6	7	8	9	10	11	12	13	14	15	16		
1		99.5	99.5	99.5	99.1	99.1	98.6	98.6	98.6	98.6	98.6	95.9	95.5	94.6	94.4	95.5	1	Buffalo A
2	0.5		99.1	99.1	98.6	99.5	99.1	99.1	99.1	99.1	98.1	95.5	95.0	94.1	93.9	95.0	2	Buffalo B
3	0.5	0.9		99.1	99.5	98.6	98.2	98.2	98.2	98.2	98.1	95.5	95.0	94.1	93.9	95.0	3	Buffalo C
4	0.5	0.9	0.9		99.5	98.6	98.2	98.2	98.2	98.2	98.1	95.5	95.0	94.1	93.9	95.0	4	Buffalo D
5	0.9	1.4	0.5	0.5		98.2	97.7	97.7	97.7	97.7	97.7	95.0	94.6	93.7	93.4	94.6	5	Buffalo E
6	0.9	0.5	1.4	1.4	1.8		99.5	99.5	99.5	99.5	97.7	95.0	94.6	93.7	93.4	94.6	6	Buffalo F
7	1.4	0.9	1.8	1.8	2.3	0.5		99.1	99.1	99.1	97.2	94.6	94.1	93.2	93.0	94.1	7	Buffalo G
8	1.4	0.9	1.8	1.8	2.3	0.5	0.9		99.1	99.1	97.2	94.6	94.1	93.2	93.0	94.1	8	Buffalo H
9	1.4	0.9	1.8	1.8	2.3	0.5	0.9	0.9		99.1	97.2	95.5	95.0	94.1	93.9	95.0	9	Buffalo I
10	1.4	0.9	1.8	1.8	2.3	0.5	0.9	0.9	0.9		97.2	94.6	94.1	93.2	93.0	94.1	10	Buffalo J
11	0.0	0.5	0.5	0.5	0.9	0.9	1.4	1.4	1.4	1.4		94.8	94.4	93.9	93.0	94.4	11	Buffalo K
12	4.2	4.7	4.7	4.7	5.1	5.1	5.6	5.6	4.7	5.6	3.9		99.5	98.6	98.6	99.5	12	α_{S2}-CN A
13	4.7	5.1	5.1	5.1	5.6	5.6	6.1	6.1	5.1	6.1	4.4	0.5		98.2	98.1	99.1	13	α_{S2}-CN B
14	5.6	6.1	6.1	6.1	6.6	6.6	7.1	7.1	6.1	7.1	4.9	1.4	1.8		97.2	98.2	14	α_{S2}-CN C
15	4.4	4.9	4.9	4.9	5.4	5.4	5.9	5.9	4.9	5.9	4.0	0.0	0.5	1.4		98.1	15	α_{S2}-CN D
16	4.7	5.1	5.1	5.1	5.6	5.6	6.1	6.1	5.1	6.1	4.4	0.5	0.9	1.8	0.5		16	α_{S2}-CN E
	1	2	3	4	5	6	7	8	9	10	11	12	13	14	15	16		

Figure S1. Percent identity/divergence among the variant sequences of buffalo α_{S2}-CN and *Bos* genus α_{S2}-CN. Data above the diagonal line represents the percent identity and below the diagonal line represents the sequence divergence.

```
                   10        20        30        40        50        60        70        80
                    |         |         |         |         |         |         |         |
Buffalo A  KHTMEHVSSSEESIISQETYKQEKNMAIHPSKENLCSTFCKEVIRNANEEEYSIGSSSEESAEVATEEVKITVDDKHYQK
           hhhhhhcccchhhhhhhhhcccccceeeccccchhhhhhhhhhhhccchhheeccccchhhhhcchhhhheehhhhhhhh
 Cattle A  KNTMEHVSSSEESIISQETYKQEKNMAINPSKENLCSTFCKEVVRNANEEEYSIGSSSEESAEVATEEVKITVDDKHYQK
           hhhhhhcccchhhhhhhhhcccccceeecccccccchhhhhhhhhhhhhhhecccccchhhhhcchhhhheehhhhhhhh
                   90       100       110       120       130       140       150       160
                    |         |         |         |         |         |         |         |
Buffalo A  ALNEINQFYQKFPQYLQYLYQGPIVLNPWDQVKRNAVPITPTLNREQLSTSEENSKKTVDMESTEVFTKKTKLTEEDKNR
           hhhhhhhhhhhhhhhhhhhcccceeecccccccccccceeccccccchhccchhcccheeeecccccccccccchhhhhhhh
 Cattle A  ALNEINQFYQKFPQYLQYLYQGPIVLNPWDQVKRNAVPITPTLNREQLSTSEENSKKTVDMESTEVFTKKTKLTEEEKNR
           hhhhhhhhhhhhhhhhhhhtccceeecccccccccccceecccccccheccchhcccceeeehcccccccchhhhhhhhhhh
                  170       180       190       200
                    |         |         |         |
Buffalo A  LNFLKKISQHYQKFAWPQYLKTVYQYQKAMKPWTQPKTNVIPYVRYL
           hhhhhhhhhhhhhcccchhhhhccchhhhccttcccccccceecceee
 Cattle A  LNFLKKISQRYQKFALPQYLKTVYQHQKAMKPWIQPKTKVIPYVRYL
           hhhhhhhhhhhhtcccchhhhhcccchhcccttccccceeeeeccce
```

Figure S2. Predicted secondary structure of mature peptide of α_{S2}-CN variant A in buffalo and cattle. Alpha helices, extended strands, beta turns, and random coils are indicated with the h, e, t, and c, respectively.
